# Impact of electric cardioversion on platelet activation

**DOI:** 10.1371/journal.pone.0250353

**Published:** 2021-04-22

**Authors:** Harald Haidl, Johanna Gaugler, Gerhard Cvirn, Hildegard Jasser-Nitsche, Wolfgang Schwinger, Sina Pohl, Egbert Bisping, Siegfried Gallistl, Axel Schlagenhauf

**Affiliations:** 1 Division of General Paediatrics, Department of Paediatrics and Adolescent Medicine, Medical University of Graz, Graz, Austria; 2 Division of Physiological Chemistry, Otto Loewi Research Center, Medical University of Graz, Graz, Austria; 3 Division of Paediatric Haematology/Oncology, Department of Paediatrics and Adolescent Medicine, Medical University of Graz, Graz, Austria; 4 Division of Cardiology, Department of Internal Medicine, Medical University of Graz, Graz, Austria; Institut d’Investigacions Biomediques de Barcelona, SPAIN

## Abstract

**Introduction:**

Atrial fibrillation (AF) comes along with high risk of stroke. This risk continues even after re-establishing sinus rhythm with cardioversion. Aim of this study is to evaluate the contribution of electric cardioversion (EC) to platelet activation and procoagulatory tendency.

**Methods:**

Extent of platelet activation before and after electric cardioversion was quantified using flow cytometry, impedance aggregation measurements with Multiplate®, and quantification of serum levels of platelet factor 4 (PF4) and ß-thromboglobulin (ß-TG) in patients with AF (N = 10).

**Results:**

No significant differences were observed in any of the measured parameters comparing the values from before and after cardioversion. Geometric means of P-selectin expression and integrin αIIbβ3 activation were 0.27 (+/- 0.07) and 2.30 (+/- 2.61) before EC and 0.28 (+/- 0.17) and 1.67 (+/- 1.82) after EC. Levels of ß-TG were 110.11 ng/ml (+/- 3.78) before and 110.51 ng/ml (+/- 2.56) after EC, levels of PF4 were 35.64 ng/ml (+/- 12.94) before and 32.40 ng/ml (+/- 4.95) after EC. Platelet aggregation triggered with adenosine diphosphate (ADP), arachidonic acid, collagen, Ristocetin, or thrombin receptor activating peptide (TRAP) revealed results within the normally expected ranges without significant changes before and after EC.

**Discussion:**

Electric cardioversion has no influence on platelet activation markers which is in agreement with other studies reporting electrical cardioversion to be safe.

## Introduction

Atrial fibrillation (AF) is associated with a high risk of stroke. AF leads to hypercoagulability via different mechanisms resulting in an increased risk for thrombus formation in the left atrium, leading—in case of disruption—to arterial embolism. This high risk for thromboembolism is the reason why anticoagulant therapy is a standard treatment of AF according to the CHA_2_DS_2_-VASc score [1].

Treatment options to achieve rhythm control and re-establish a sinus rhythm are pharmacological cardioversion (PC) or electrical cardioversion (EC). Once a normal heart rhythm is established, however, the risk for embolism is not averted immediately. Therefore, anticoagulation is maintained for at least 4 weeks after cardioversion. The usually given explanation for this prolonged procoagulant tendency is atrial stunning. Atrial stunning is a transient mechanical dysfunction of the left atrium and left atrial appendage with lower blood flow velocities after cardioversion [2]. The risk for post-cardioversion thromboembolic complications is even higher in certain subgroups of patients who had cardioversion due to AF with a duration < 48 h and had no anticoagulation prescribed as showed by Airaksinen et al. [3].

EC is performed as electric shock synchronized with electrocardiography. If successful, depolarisation of myocardial cells leads to a restored sinus rhythm [4]. Electric current not only has an impact on the heart conduction system but is also used therapeutically, e.g. for electric convulsion therapy or electrosurgery.

The influence of electric current on haemostasis has been postulated by different groups as thrombosis occurred in certain body regions after electric injury, even when these were not directly passed by the current [5–7].

In vitro, experimental works were done to enlighten the possible influence of electricity on haemostasis. Ulrich et al. focused on endothelial cells that were exposed to an electric field and found alterations in plasminogen activator inhibitor-1, tissue plasminogen activator, and tissue factor [8]. Frelinger showed that pulse electric field (PEF) stimulation of platelet-rich plasma is at least as effective as stimulation with thrombin receptor activating peptide (TRAP) or bovine thrombin [9]. In 2018, Hardy et al. presented the development of an interdigitated microelectronic bandage that augments haemostasis and clot formation [10].

Regarding the thrombotic risk in connection with AF, cardioversion and electric injuries at the one hand, and the procoagulant features of activated platelets [11] at the other, we wanted to test our hypothesis: electric current during the procedure of cardioversion activates platelets which may have an additional influence in generating thrombosis in post-cardioversion patients atrial stunning.

## Materials and methods

Study protocol (S1 File) was authorized by the local ethics committee of the Medical University of Graz (EK-Nr. 27–155 ex 14/15). All procedures performed in the study involving human participants were in accordance with the 1964 Helsinki declaration and its later amendments.

In this study, platelet activation was measured before and after the procedure of EC.

Patients with AF (N = 10) undergoing elective direct current cardioversion at the cardiology`s daycare unit of the Medical University of Graz were asked to participate in the study and gave their written informed consent. Recruitment took place between May 2015 and October 2017, analyses were performed in our in-house research laboratories. Different from the protocol planned none of the patients could be recruited for a third blood draw 24 h after EC. Patient details are given in Table 1. All patients had anticoagulant treatment. Exclusion criteria were the intake of antiplatelet therapy. For the procedure patients were sedated using midazolam and fentanyl.

**Table 1:** Patient details.

patient	sex	age (yrs)	EC successful	tries	energy (J)	anticoagulation
# 1	m	25,4	y	1 x	50	Edoxaban
# 2	m	64,2	y	1 x	200	Dabigatran
# 3	m	61,2	y	1 x	200	Dabigatran
# 4	f	72,5	y	1 x	200	Rivaroxaban
# 5	f	44,0	n	2 x	150, 270	Phenprocoumon
# 6	m	47,8	y	3 x	n.d.	Rivaroxaban
# 7	m	62,8	y	1 x	70	Phenprocoumon
# 8	m	43,7	n	2 x	150, 200	Rivaroxaban
# 9	f	73,6	y	1 x	200	Rivaroxaban
# 10	m	76,5	y	2 x (ICD)	40, 40	Rivaroxaban

EC successful states whether cardioversion worked: yes (y) or no (n); tries gives number of EC tries on this day; energy: used energy in Joule (J) that was given for cardioversion; n.d.: not documented.

Blood was drawn into precitrated tubes (Vacutainer®) and Hirudin containing tubes (Sarstedt®) at admittance and processed immediately. A second blood draw was scheduled one hour after EC.

For flow cytometry analyses, blood was diluted 1:10 with PBS-buffer (Dulbecco`s Phosphate Buffered Saline; Gibco). 50 μl portions of this solution were taken for further steps: CD41-PC7 antibody (Beckman Coulter) was added in order to label platelets.

For P-selectin expression and integrin αIIbβ3 activation, CD62P-PE (BD Biosciences) and PAC-1-FITC (BD Biosciences) antibodies were used, respectively. Isotype control was IgG1 κ-PE (BD Biosciences) for CD62P. 10 μg/vial RGDS peptide (Sigma Aldrich) was used additionally to PAC-1 antibodies to obtain a negative control for PAC-1. A sample from a healthy adult was stimulated with collagen (3.2 μg/ml) before analysis to obtain a positive control. All reagents were used according to manufacturer’s instruction. Incubation for 20 minutes in the dark was followed by a fixing step with 1% formaldehyde at 8°C. Flow cytometry measurement was performed using a Beckman Coulter FC500. Data analysis was performed with FlowJo V10 (BD Biosciences). Obtained geometric means were used for statistical analyses.

In a subgroup of 5 patients, whole blood impedance aggregation measurements were performed by means of Multiplate® (Roche Diagnostics): whole blood anticoagulated with Hirudin was analysed after activation with adenosine diphosphate (ADP, 6.5 μM), arachidonic acid (0.5 mM), collagen (3.2 μg/ml), Ristocetin (0.77 mg/ml) or thrombin receptor activating peptide (TRAP, 32 μM) according to manufacturer’s instruction and the area under the curve was calculated.

In a second subgroup of 5 patients, beta-thromboglobulin (ß-TG), also known as Chemokine (C-X-C motif) ligand 7 (CXCL7), and platelet factor 4 (PF4), also known as Chemokine (C-X-C motif) ligand 4 (CXCL4) were measured by ELISA (Abcam) in platelet-poor plasma.

SPSS Vers. 25 was used for statistical analyses. Results of all measurements after EC were evaluated for differences to baseline levels before EC using Students t-test. P-values <0.05 were considered statistically significant. Data are presented as mean ± standard deviation. Figures were grafted with GraphPad 6.0.

## Results

Multiplate® analyses revealed no significant differences in platelet aggregation before and after EC. Table 2 shows means (SEM) of AUC values.

**Table 2:** Platelet aggregation.

Aggregation trigger	Pre-EC	Post-EC	p
ADP	89.6 (13.4)	82.6 (8.4)	0.436
ASPI	85.8 (23.1)	97.6 (9.5)	0.575
Collagen	84.0 (8.1)	97.6 (8.8)	0.271
Risto	104.4 (11.6)	92.2 (11.4)	0.433
TRAP	121.0 (18.6)	124.2 (17.8)	0.178

Means of the area under the curve (AUC) values of Multiplate® aggregation measurements (SEM) in arbitrary units. For activation of platelets, adenosine diphosphate (ADP), arachidonic acid (ASPI), collagen, Ristocetin (Risto), or thrombin receptor activating peptide (TRAP) was used. P-values of t-test were calculated for each agonist.

Measurement of ß-TG and PF4 as soluble marker of platelet activation showed no significant difference before and after EC; we found 110.11 ng/ml (+/- 3.78) before and 110.51 ng/ml (+/- 2.56) after EC of ß-TG (p = 0.865), and 35.64 ng/ml (+/- 12.94) before and 32.40 ng/ml (+/- 4.95) after EC of PF4 (p = 0.584), respectively.

**Fig 1:**
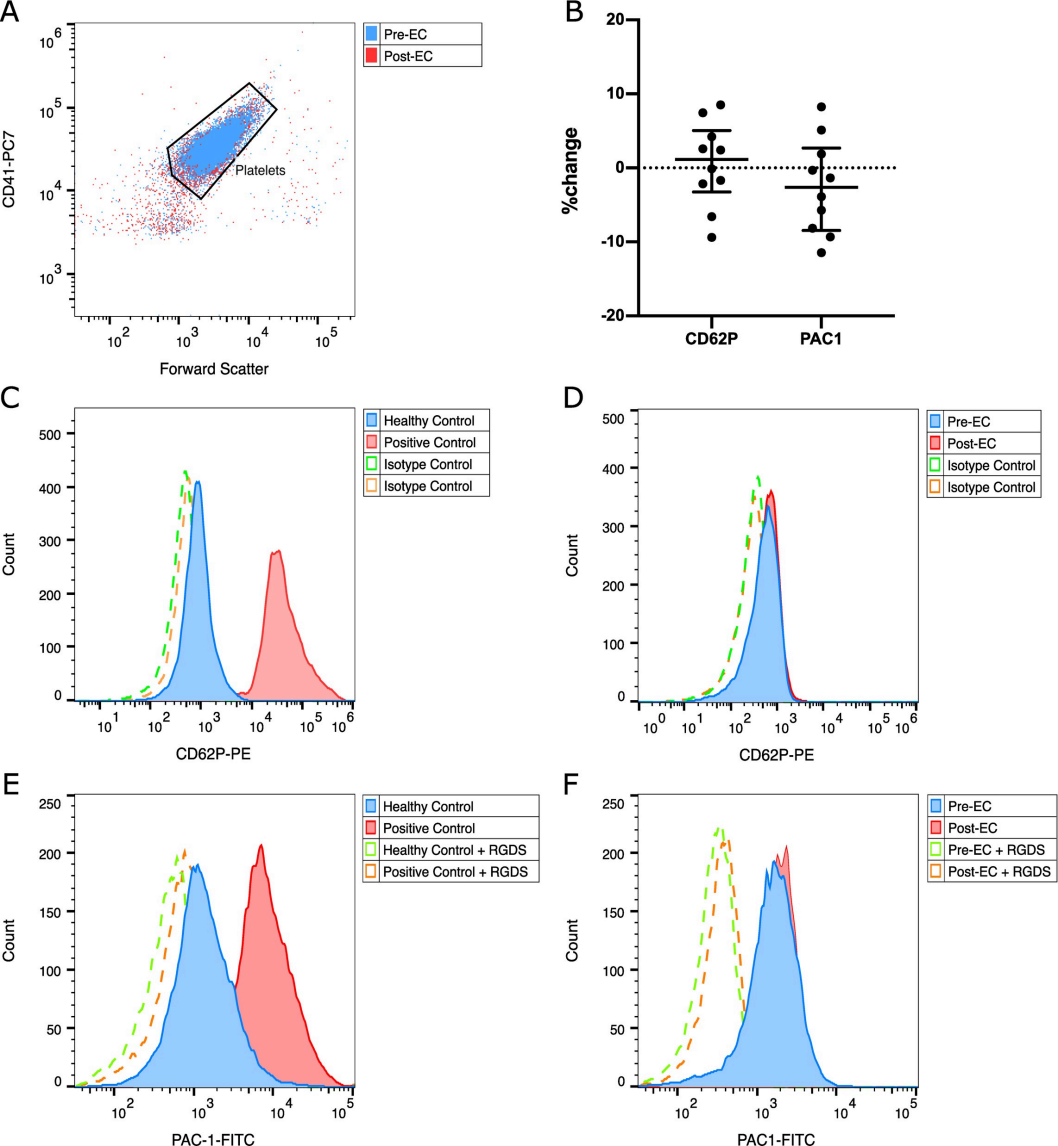
Flow cytometric analysis of expression of CD62P and activated integrin αIIbβ3 before and after EC. (A) Gating strategy based on the pan-platelet-marker CD41-PC7, (B) relative changes of CD62P and activated integrin αIIbβ3 of each patient, (C) CD62P expression of a non-stimulated healthy control (blue) and after in vitro stimulation with collagen (red), (D) representative histogram of CD62P expression of a patient before (blue) and after (red) EC, (E) activated integrin αIIbβ3 expression of a non-stimulated healthy control (blue) and after in vitro stimulation with collagen (red), (F) representative histogram of activated integrin αIIbβ3 expression before (blue) and after (red) EC; (C-F) dashed lines depict isotype controls for CD62P or negative controls for PAC-1.

Comparing the activation markers P-selectin and activated integrin αIIbβ3 on the platelet surface with flow cytometry we found no significant difference before and after EC. Fig 1 shows our gating pattern and representative graphs. Geometric means of expression of P-selectin were 0.27 (+/- 0.07) before and 0.28 (+/- 0.17) after EC (p = 0.771), those of activated integrin αIIbβ3 were 2.30 (+/- 2.61) before and 1.67 (+/- 1.82) after EC (p = 0.254).

## Discussion

We found no statistically significant changes in platelet activation markers after EC in our patient studies, neither in flow cytometry analyses nor in ß-TG and PF4 levels. Platelet reactivity determined by Multiplate® aggregation measurements was unaltered by the procedure.

Some considerations would have argued against our hypothesis: pharmacological cardioversion also involves the risk of thromboembolism. However, Airaksinen describes a higher risk after electrical than after pharmacological cardioversion alone without peri-procedural anticoagulation (incidence rates: 1:123 vs. 1:332) but found it not statistically significant [3]. The RHYTHM-AF study found no significant differences in the incidence of thromboembolic events comparing electrical and pharmacological cardioversion and declares both modes as similarly safe [12]. Khan described no thromboembolic side effect in a review about amiodarone for cardioversion in recent onset AF [13].

There is only a small amount of blood present between the CV paddles and the electrical pulses are short. Thus, even if platelet activation takes place locally, we presume that these platelets distribute quickly via blood flow and have therefore no systemic effects. Transient reversible platelet activation may also be possible, but cannot be tested with our experimental setup. In any case, if it were reversible within 1 hour, it would not have any systemic effects.

All of our patients received anticoagulation according to guidelines. This treatment may protect platelets from activation by inhibition of thrombin generation. Hence, thrombin mediated platelet activation during EC in patients without anticoagulation cannot be fully ruled out.

In our study, we focused on platelet activation. A local effect of electrical current on endothelium of the heart or myocardial cells that act as procoagulatory triggers cannot be excluded.

We conclude that significant platelet activation via electric current applied within the procedure of EC cannot be observed making a relevant contribution to the elevated risk of thrombosis after AF unlikely.

## Supporting information

S1 File Study protocol. Study protocol approved by the local ethics committee in German and English transcript.
(DOCX)

S1 Dataset (ZIP)
