# Supplementary material for: Impact of electric cardioversion on platelet activation
Source: PLoS One. 2021 Apr 22;16(4):e0250353. doi: 10.1371/journal.pone.0250353 (PMC8061933; doi:10.1371/journal.pone.0250353)
Supplement: S1 File — Study protocol approved by the local ethics committee in German and English transcript. (DOCX) [file pone.0250353.s001.docx]

**Studienprotokoll**

(Version 2.0, 10.02.2015)

**Pilotstudie: Untersuchung über die Aktivierung von Thrombozyten durch**

**Kardioversion**

**Ziel der Studie:** Eine Vorhofflimmerarrhythmie ist eine häufige Erkrankung und die elektrische Kardioversion eine gängige Behandlungsmethode zur Wiedererlangung eines Sinusrhythmus. Da bekannt ist, dass der Zustand des Vorhofflimmerns mit einem erhöhten Aufkommen von thrombembolischen Geschehen, vor allem cerebralen Insulten, vergesellschaftet ist, ist eine Antikoagulation dieser Patienten obligat. Dieses erhöhte Schlaganfallrisiko bleibt aber auch weiter bestehen für die ersten Wochen nach erfolgter Kardioversion bei schon wieder bestehendem Sinusrhythmus. Als Ursache dafür wird das sogenannte „atrial stunning“ angenommen: Der Vorhof hat durch das vorherige Flimmern noch nicht seine ursprüngliche Kontraktilität wiedererlangt. Dadurch kommt es zu einem mangelnden Blutfluss im Bereich des Vorhofes, was wiederum die Entstehung von Thromben begünstigt. Zum Beitrag von Thrombozyten bei der Entstehung solcher Thromben gibt es bis dato wenig Daten. Es stellt sich uns jedoch die Frage, ob durch das Anlegen eines elektrischen Stromes, wie er für die Kardioversion verwendet wird, es auch zu einer Affektion von

Blutbestandteilen kommt. Das Ziel unserer Studie ist die Untersuchung einer möglichen

Aktivierung von Thrombozyten durch elektrischen Strom, die in weiterer Folge an der

Entstehung von Gerinnseln beteiligt sein könnten.

**Patienten:** Es handelt sich um eine Pilotstudie. Als Probanden kommen Patienten in Frage, bei denen eine elektrische Kardioversion geplant ist. Die Rekrutierung erfolgt an der Universitätsklinik für Kardiologie. Diese werden gefragt, ob sie bereit sind für diese Studie vor und zweimal nach Kardioversion (eine Stunde und 24 – 48 h danach) je 6 ml Blut zu spenden.

Einschlusskriterien: 1. Indikation zur elektiv geplanten elektrischen Kardioversion bei

persistenter Vorhofarrhythmie. 2. Lebensalter von 19 bis 80 Jahren. 3. Unterschriebene

Einwilligungserklärung.

Ausschlusskriterien: fehlende Einverständniserklärung

**Nutzen-Risiko-Darstellung:** Da die Patienten im Rahmen ihrer Behandlung einen i.v.-Zugang benötigen, ist initial keine zusätzlich invasive Maßnahme nötig. Lediglich tagesklinisch behandelte Patienten, die für eine dritte Blutabnahme kommen, haben das Nebenwirkungsrisiko einer zusätzlichen Blutabnahme. Das entnommene Blutvolumen ist für einen Erwachsenen nicht relevant und an der Behandlung der Erkrankung selbst wird sich nichts ändern. Ein unmittelbarer Nutzen für den Teilnehmer ist nicht zu erwarten. Der Nutzen liegt in einem besseren Verständnis der hämostaseologischen Vorgänge bei Kardioversion.

**Methodik:** Nach erfolgter Aufklärung und Einholen einer Einverständniserklärung wird im Rahmen der regulären Blutabnahmen vor Kardioversion 3 ml Citratblut und 3 ml Blut versetzt mit Hirudin mit abgenommen. Aus dem Citratblut werden mittels FACS Aktivierungsmarker (P-Selektin und PAC-1) auf der Thrombozytenoberfläche gemessen und mit Werten 1 Stunde und 24 – 48 Stunden nach der Kardioversion verglichen. Mit den weiteren 3 ml wird eine

Thrombozytenaggregationsmessung mittels MultiPlate© durchgeführt und ebenfalls die Werte vor und nach Kardioversion verglichen.

**Statistik:** Fallzahlberechnung: Da es sich um eine Pilotstudie handelt und es keine Vordaten gibt, kann keine Fallzahlberechnung durchgeführt werden und die Pilotstudie wird mit einer

Probandenanzahl von 20 Personen durchgeführt.

Zielgrößen: Die Hauptzielgröße ist der PAC-1-Oberflächenmarker, Nebenzielgrößen sind p-

Selektin und die Parameter der Thrombozytenaggregation.

Null-Hypothese: Es ergibt sich keine Änderung der Hauptzielgröße durch Kardioversion.

Datenspeicherung: Die Speicherung erfolgt anonymisiert in einer Excel-Tabelle.

Datenanalyse: Die Analyse erfolgt mittels IBM SPSS Statistics 22©. Unterschiede zwischen

den Gruppen werden mit Hilfe einer ANOVA und nachfolgenden Post-hoc tests analysiert.

**Study protocol - transcript**

**(Version 2.0, 10.02.2015)**

**Pilot trial: Examination of platelet activation during cardioversion**

**Aim:** Atrial fibrillation (AF) is a common disease and electric cardioversion one of the common treatment to re-achieve a sinus rhythm. AF is often associated with stroke, so anticoagulation is – with exclusions – mandatory. The elevated risk for stroke persists even after cardioversion with re-established sinus rhythm. As reason for this “atrial stunning” is the accepted explanation: Contractibility of atrial myocardium after cardioversion is still reduced. Due to reduced blood flow in the atrium development of thrombotic formations is promoted.

A contribution of platelets to this thrombotic risk was not an issue by now. Our question in this context is whether electric current influences blood components. In this study a potential activation of platelets by electricity which possibly facilitates development of thrombosis.

**Patients:** This is a pilot trial. Patients from cardiology department undergoing elective electric cardioversion are asked to participate and donate 3 times 6 ml of blood (before cardioversion, 1 and 24-48 h after cardioversion).

Inclusion criteria: 1. Persistent atrial fibrillation and indication for cardioversion. 2. Age from 19 – 80 years. 3. Written informed consent.

Exclusion criteria: Lack of consent.

**Benefit-risk-statement:** For the cardioversion patients need an intravenous line which can be used for blood draws; the last blood draw – if participants are outclinic patients – would be an extra stich including risk of adverse events. The amount of blood drawn for this study is negligible. Treatment of AF is not affected by study-correlated procedures.

There is no benefit for the individual participants. Benefit lays in better understanding of hemostasis under cardioversion.

**Methods:** After information and signing consent 6 ml blood (3 ml citrated whole blood and 3 ml mixed with Hirudin) is drawn together routine testing. Citrated blood is used for FACS analysis (P-selectin and PAC-1). Results are compared with measurements 1 and 24- 48 hours after cardioversion. Platelet aggregation from Hirudin blood is measured with MultiPlate^©^ and results are compared before and after cardioversion.

**Statistics:** Calculating for number of cases: As pilot trial and without preliminary data a calculation can not be done. The pilot trial is planned with 20 participants.

Primary variable is PAC-1 platelet surface marker; supplementary variables are P-selectin and parameters of platelet aggregation.

Null hypothesis: There is no change in primary variable provoked by cardioversion.

Data management: Anonymized saving of data in an excel table.

Data analysis: Analysis is performed with IBM SPSS 22. Differences will be tested with ANOVA followed by post-hoc-tests.
